# Genome-Wide Association Study of Body Weight Traits in Texel and Kazakh Crossbred Sheep

**DOI:** 10.3390/genes15121521

**Published:** 2024-11-27

**Authors:** Sheng Wang, Mingjun Liu, Huiguo Zhang, Sangang He, Wenrong Li, Long Liang

**Affiliations:** 1 College of Mathematics and System Science, Xinjiang University, Urumqi 830000, China; swang@stu.xju.edu.cn; 2 Key Laboratory of Animal Biotechnology of Xinjiang, Key Laboratory of Genetics Breeding and Reproduction of Grass Feeding Livestock, Minisitry of Agriculture and Rural Affairs of the People’s Republic of China, Institute of Biotechnology, Xinjiang Academy of Animal Science, Urumqi 830000, China; liwenrong@xjaas.net (W.L.); dl861218@163.com (L.L.); 3 Institute of Animal Husbandry and Veterinary, Zhejiang Academy of Agricultural Sciences, Hangzhou 310021, China; hesg@zaas.ac.cn

**Keywords:** GWAS, post-GWAS, genetic parameters, body weight, Texel-Kazakh crossbred sheep

## Abstract

**Background:** Originating from the cold and arid regions of northwestern China, Kazakh sheep are dual-purpose breeds optimized for both meat and fat production. In contrast, Texel sheep are internationally recognized for their high-quality meat and exceptional flavor. Previous studies have indicated that the hybrids of Texel and Kazakh sheep exhibit significant quality advantages. Additionally, body weight is a crucial indicator of sheep production performance, directly correlating with meat yield and economic returns. **Objective:** This study aims to identify genetic variations and related genes associated with the body weight traits of hybrid lambs, thereby revealing their genetic mechanisms. **Methods:** This study genotyped hybrid lambs using a 50K chip and performed rigorous quality control on both genotypic and phenotypic data. The traits examined include body weight traits of lambs at various stages such as birth, pre-weaning, and post-weaning. Various genome-wide association study (GWAS) models were utilized to analyze the association between lamb body weight traits and genetic markers. The study then employed an Ensemble-like GWAS (E-GWAS) strategy to integrate these models, achieving a stable list of SNPs, rather than a mere aggregation. Multiple annotation databases were consulted to further investigate the mechanisms by which genetic markers affect body weight traits. All study results were validated through an extensive literature review. **Results:** Analyses with multiple statistical models revealed that 48 SNPs were significantly associated with body weight traits. The annotation process identified 24 related genes (including 4 unknown genes) and 9 quantitative trait loci (QTLs). Additionally, 6 Gene Ontology (GO) terms and 22 Kyoto Encyclopedia of Genes and Genomes (KEGG) pathways were determined. **Conclusions:** This study identified key genes and pathways in the body weight traits of hybrids between Texel and Kazakh sheep, enhancing our understanding of their genetic mechanisms.

## 1. Introduction

Body weight is critical for meat production and economic returns in sheep breeding [1]. Local sheep breeds often grow slowly and yield insufficient meat, not meeting market demands. Breeders address this by crossing them with high-yield foreign breeds, which maintains the adaptability of the local breeds while enhancing growth and meat production through moderate heterosis. Texel sheep, originating from Dutch Texel Island in the mid-19th century, are celebrated for their premium meat. In contrast, Kazakh sheep from Xinjiang in China exhibit distinctive characteristics, including a brown coloration, elongated ears, expansive backs, and robust limbs. Their tails, surrounded by fat, enhance environmental resilience and disease resistance, rendering them well-suited for harsh grazing environments [2]. Hybrids of Texel rams and Kazakh ewes show significant improvements in daily weight gain, carcass weight, resilience, and adaptability [3]. This crossbreeding also compensates for the low fertility of Kazakh sheep, thus boosting overall production [4]. Despite the lack of detailed studies on body weight traits of Texel-Kazakh hybrids, their evident potential suggests a need for further genetic and performance investigations.

Genome-wide association studies examine genomic markers to pinpoint genetic variations linked to specific traits [5]. Utilizing genotyping tools, these studies accurately identify markers associated with pivotal economic traits in the sheep genome, such as growth characteristics, reproductive capabilities, and dairy product quality. This identification facilitates substantial advancements in genetic enhancement and breed optimization for sheep [6]. Li et al. utilized microsatellite markers to assess the genetic variability within the Texel-Kazakh sheep crossbreeds and its correlation with production traits [7]. Currently, research on the genetic attributes of Texel-Kazakh sheep hybrids remains scant.

GWAS has spurred the development of various models and software tools like Plink [8], GCTA [9], and GAPIT [10] to simplify their application. GAPIT v3.0 [10], an R package, integrates advanced GWAS models surpassing those in Plink [8] and GCTA [9] and offers robust visualization capabilities. GAPIT incorporates over ten models, including the basic *t*-test model, which uses a general linear model (GLM) [11] to examine the relationship between phenotypes and genetic markers, factoring in population structure (Q matrix) to minimize false-positive results. The mixed linear model (MLM) [12] builds on this by including genetic effects as random cofactors within a kinship-controlled variance structure (K matrix), though it risks introducing confounding effects, and some genes detectable under GLM remain elusive under MLM. To overcome this, the compressed MLM (CMLM) [13] replaces individual genetic and kinship effects with group-based effects, optimized via maximum likelihood, enhancing statistical power and enabling transitions between GLM and MLM depending on the group size. The multiple loci mixed model (MLMM) [14] advances this by integrating associated markers as cofactors, utilizing stepwise regression and maintaining constant Q and K matrices. The SUPER [15] method further addresses confounds by deriving the K matrix from associated markers. FarmCPU [16] employs a fixed-effect approach, interchanged with a model of random effects, to select markers based on kinship from associated markers and a bin strategy to prevent marker overlap. Lastly, BLINK boosts statistical power in large datasets by using linkage disequilibrium (LD) methods to eliminate closely linked markers, approximating maximum likelihood with Bayesian Information Content in its fixed-effect model [17].

Post-GWAS facilitate enhanced comprehension of significant SNPs identified through GWAS models. As GWAS methodologies advance, the integration of diverse approaches merits careful consideration. Recently, Li et al. [18] introduced the E-GWAS strategy for post-GWAS, which combines results from multiple GWAS methods. This approach reduces the rate of false positives and enhances the consistency of true positive rates. Furthermore, numerous databases enhance the understanding of significant genetic markers. Platforms such as the National Center for Biotechnology Information (NCBI) [19] website and the Animal QTL Database [20] provide comprehensive annotations for genes and QTLs. Researchers have developed various databases to categorize gene functions and elucidate their roles and regulatory mechanisms within organisms. Notably, the GO and KEGG databases stand out. GO analysis elucidates the roles of genes within biological processes (BPs), cellular components (CCs), and molecular functions (MFs). Concurrently, KEGG analysis furnishes comprehensive details on the metabolic and signal transduction pathways involving these genes, thereby enhancing understanding of their biological implications. Additionally, the clusterProfiler v4.0 [21] R package integrates support for multiple bioinformatics databases, including GO and KEGG, thereby facilitating streamlined enrichment analysis.

The aim of this study was to determine the genetic factors influencing body weight in Texel and Kazakh sheep hybrids by applying various GWAS models. These models helped identify specific SNPs associated with body weight traits. The implementation of post-GWAS was then carried out to investigate the specific impacts of these significant SNPs on the body weight of the hybrid offspring.

## 2. Materials and Methods

### 2.1. Ethics Statement

All experimental procedures adhered rigorously to the guidelines outlined in the Guide for the Care and Use of Laboratory Animals by the Xinjiang Academy of Animal Sciences, China. Ethical approval for all experimental protocols was obtained from the Ethics Committee of the Xinjiang Academy of Animal Sciences (Approval No. XJAAS-AE-20212808).

### 2.2. Animals and Samples

This research procured all experimental animals from the Kazakh Sheep Breeding Base at Zhaosu Stud Farm in Zhaosu County, Yili Kazakh Autonomous Prefecture, Xinjiang Uyghur Autonomous Region, China, with data provided by the Xinjiang Academy of Animal Science. Based on previous preliminary research [3,4], Texel sheep served as the paternal stock to the maternal Kazakh stock, generating a first-generation (F1) crossbreed. This F1 generation was subsequently bred with Texel sheep to produce a second-generation (F2) crossbreed. In spring 2021, all newborn lambs were grouped into seven units based on four distinct crossbreeding combinations and managed in confinement alongside their mothers. The facility adhered to strict standardized feeding and management protocols to ensure consistent care for all animals.

Blood samples were obtained from individual sheep via jugular venipuncture, with each sample comprising no less than 3 mL. Upon collection, these samples were directly transferred into vacuum tubes containing ethylenediaminetetraacetic acid (EDTA) to inhibit coagulation and maintain the integrity of the blood, essential for DNA extraction (Compsen Biotechnology Co., Ltd., Beijing, China). To retard metabolic activity and avert DNA degradation, the samples were subsequently preserved at 4 °C. Genotyping analyses were performed utilizing the 50K SNP Array (Compsen Biotechnology Co., Ltd., Beijing, China), with reference to the Oar v4.0 (GCF 000298735.2) ovine genome assembly available from NCBI [19].

Lamb weights and measurement dates were meticulously documented on a regular basis. In the initial stages, measurements were taken frequently, but as time progressed, the interval between measurements gradually increased. This dataset contained 18 weight measurement records. To ensure data accuracy, lambs must fast for 12 h before measurement, and their weight was measured using an automatic scale, recorded in kilograms and accurate to one decimal place by skilled technicians. Additionally, the dataset included information on the lambs’ gender, birth type, and hybrid group.

### 2.3. Genotyping Analysis and Data Quality Control

The study initiated with quality control on genotype data using Plink v1.90 [8], involving the removal of duplicate SNPs and datasets where SNP and sample missing rates exceeded 1% each. Missing genotype data were imputed using Beagle v5.4 [22], achieving a complete dataset. Further, Plink v1.90 [8] was utilized to exclude SNPs with a minimum allele frequency of less than 0.05 or below the Hardy-Weinberg equilibrium test threshold of $10^{-6}$. To prevent false positives arising from high kinship, analysis excluded parent-offspring and sibling pairs.

Phenotypic data quality control was conducted using R v4.3.2 [23], encompassing data correction, outlier handling, and normal distribution testing. The average daily weight gain method was used to correct phenotypic data. Researchers adjusted weight records based on specific constant age days to establish a fair benchmark for comparison. Notable variations were detected in the growth rates of lambs, which generally declined following weaning. Therefore, to ensure data accuracy, records from before and after weaning were not used concurrently for the same constant age day adjustments. Outliers were handled using the three standard deviation method. Specifically, data points for a given trait exceeding three times the standard deviation of that trait were removed from the dataset. Normality testing of traits was conducted using the Shapiro-Wilk method. This study investigated the body weight of crossbred sheep at various developmental stages, including birth weight (W0), pre-weaning weight (weight at 30 days (W30), weight at 60 days (W60), weight at 90 days (W90)), and post-weaning weight (weight at 180 days (W180) and weight at 360 days (W360)). These measurements reflect body weights at critical developmental stages: birth, the onset of accelerated growth, peak growth rate, weaning, six months, and one year (end of winter/early spring). The analysis ultimately included 578 samples and 51,522 autosomal SNPs.

### 2.4. Estimation of Genetic Parameters

Estimating heritability by integrating phenotypic data across multiple traits generally yields more realistic results compared to analyses limited to a single trait. This occurs because multi-trait models account for the inter-relationships among various traits, whereas single-trait models do not. Consequently, we utilized the multi-trait model provided by Hiblup v1.4.0 [24] software to estimate the heritability of each trait concurrently. In order to compare the differences between the single-trait model and the multi-trait models, we also used the single-trait model to evaluate the heritability of each trait.

To increase analytical precision, principal component analysis (PCA) was integrated with the genomic relationship matrix. This integration was essential in managing potential covariance among trait measurements arising from population stratification and hidden familial links, thus efficiently minimizing the false-positive rate. The decision to utilize the matrix of principal components generated by PCA, instead of the Q matrix, was made because constructing the Q matrix necessitates predicting the number of subgroups in genotype data—a complex and time-intensive process. Population stratification was assessed and further selection of principal components (PCs) was conducted using the method described by Patterson [25], which relies on the Tracy-Widom statistic. This approach facilitates the testing of the significance of each principal component to minimize subjective judgment. However, the Patterson method may necessitate incorporating numerous principal components into the model. Consequently, the decision was made to include only those principal components accounting for more than 1% of the variance as fixed effects in the model of interest.

Additionally, based on empirical evidence and optimal utilization of available data for managing non-genetic influences, it is recommended that these factors be integrated into models estimating genetic parameters and GWAS. In the dataset, birth weight (W0) is typically unaffected by feeding conditions. Traits W30 through W90 represent lamb weights at three distinct pre-weaning stages, whereas W180 and W360 correspond to weights at two separate post-weaning stages. Analysis suggests that the age of the ewes does not influence lamb weights at these post-weaning stages due to the cessation of lactation. Therefore, the factors detailed in Table 1 were incorporated concurrently as fixed effects.

### 2.5. GWAS Model Analysis Methods

This research investigated genome-wide associations of six different growth traits using various GWAS models from the GAPIT v3.0 [10] software package, including GLM, MLM, MLMM, FarmCPU, BLINK. The multi-model strategy enhanced both the robustness and accuracy of the analysis, while mitigating bias associated with reliance on a single model, thereby facilitating the selection of the most suitable model under specific conditions. These GWAS models utilized fixed effects identical to those applied in calculating genetic parameters, as detailed previously, encompassing principal components and the factors enumerated in Table 1.

Manhattan plots, generated by GAPIT, were used to display the chromosomal positions of SNPs and their associated significance. Additionally, this study utilized quantile-quantile (Q-Q) plots provided by GAPIT and the genome inflation factor (GIF) to identify and visualize potential biases in the statistical data. These methods help ensure result reliability and control for false positives [26]. The genome inflation factor was calculated by taking the median of the squared z-scores of the GWAS *p*-values and dividing it by the expected value from a chi-square distribution with 1 degree of freedom. The significance threshold was determined by taking the inverse of the overall count of quality-controlled SNPs across the genome. This approach mitigates false-positive issues in multiple testing via Bonferroni correction. Thus, the threshold for genome-wide significance was set at $1.94\times 10^{-5}$, which corresponds to a 4.71 on the $-log_{10}$ (*p*-value) scale, as illustrated in the Manhattan plot.

### 2.6. Integrating Multiple GWAS Models: The E-GWAS Strategy

The preceding section introduced methods for identifying significant SNPs using various GWAS techniques. The primary objective in this section is to amalgamate outcomes from multiple GWAS models to derive a robust compilation of SNPs. The E-GWAS strategy proposed by Zhou et al. [18] aptly facilitates this integration through a structured approach encompassing GWAS analysis, cross-linking, and meta-analysis. Initially, significant candidate SNPs are derived from prior GWAS analyses. In the cross-linking step, overlapping SNPs from disparate analyses are merged into a preliminary list, creating a collection of m SNPs. Subsequently, these m SNPs are analyzed as fixed effects, with the kinship matrix treated as a random effect in a mixed-effects linear model to further refine and compute *p*-values. A Pearson correlation coefficient threshold of 0.7 is applied to cluster SNP pairs that exceed this value, selecting the SNP with the lowest *p*-value in each cluster for retention. To stringently control for false positives, retained SNPs undergo re-evaluation using a mixed-effects model. Permutation testing establishes a critical threshold for significance, thereby enhancing the reliability of SNP associations in integrated GWAS outcomes. This approach utilizes the EGWAS package, initially developed by Xu (https://github.com/fangjun-xu/EGWAS, accessed on 1 November 2024).

### 2.7. Annotation Using Multiple Databases

Upon completing a GWAS, it becomes crucial to undertake gene annotation to thoroughly understand the mechanisms influencing the biological functions and traits associated with key gene regions identified. This research utilized the Oar v4.0 reference genome sourced from the NCBI [19], with annotations conducted using Bedtools v2.30.0 [27], and enhanced by resources from QTL databases [20]. Annotations were applied to all significant SNPs identified in the GWAS. Whenever the regions both upstream and downstream of a significant SNP overlapped with a recognized gene or QTL area, such SNPs were categorized as residing within that gene or QTL, thus identifying these genes as potential candidate genes or candidate QTLs. For SNPs located outside any known gene or QTL, the nearest candidate gene was identified and associated logically. Upstream and downstream intervals of significant SNPs were determined using LD analysis. PopLDdecay [28], a software tool designed for rapid LD analysis, was utilized for this analysis. To clarify the function and biological relevance of the candidate genes, we performed GO and KEGG enrichment analyses utilizing the clusterProfiler v4.0 [21] package within the R environment. A threshold for statistical significance was established at a *q*-value of *q* < 0.05 to identify biologically meaningful pathways and processes.

## 3. Results

### 3.1. Phenotypic Descriptive Statistics

**Figure 1:**
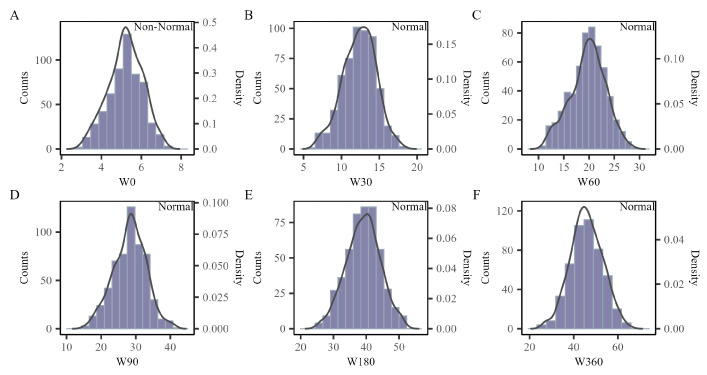
(**A**–**F**) displays the distribution of a specific trait using histograms and kernel density curves, with the outcomes of the Shapiro-Wilk test for normality (categorized as normal or non-normal) indicated in the top-right corner of each subplot.

This investigation assessed six body weight traits in 578 crossbred Texel and Kazakh sheep. The analysis included descriptive statistics such as mean, standard deviation (SD), median, minimum, maximum, skewness, kurtosis, and interquartile range (IQR), as detailed in Table 2. The Shapiro-Wilk test evaluated the normality of these continuous traits. Figure 1 presents the results, incorporating histograms and kernel density curves to depict the distribution of traits. Trait W0 exhibited slight non-normality (p≈0.03) at a 0.05 significance level, whereas the remaining traits adhered to a normal distribution.

### 3.2. Population Structure Analysis and Linkage Disequilibrium Analysis

**Figure 2:**
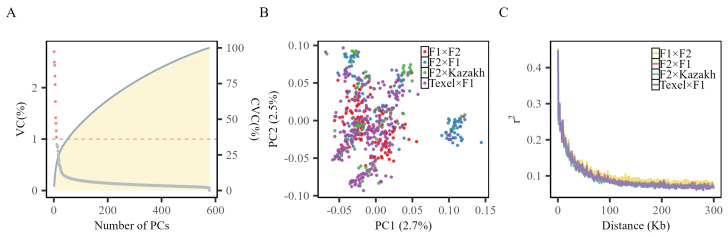
(**A**) Displays the variance of the principal components and their cumulative contribution rates. The left vertical axis shows the variance contribution (VC) rate in percentage for each principal component; the right vertical axis shows the cumulative variance contribution (CVC) rate; (**B**) depicts the division of individuals based on the first two principal components; (**C**) demonstrates how LD values for various hybrid combinations vary with changes in the distance between SNPs.

Following quality control procedures, the research utilized 51,522 SNPs for PCA. This analysis aimed to address stratification within the experimental population. Figure 2A shows the variance contribution of different number of principal components (PCs) to the data. As the count of principal components rises, the contribution rate of cumulative variance increases gradually. The scree plot Figure 2B reveals that the first principal component explains merely 2.7% of the variance, and there is no obvious boundary between different hybrid combinations, suggesting the absence of a dominant population structure significantly impacting data variability. This interpretation is supported by the Tracy-Widom statistics, indicating that incorporating the first 94 principal components into the GWAS model is advisable.

Analysis of LD in the target sheep population, as shown in Figure 2C, reveals that the offspring of different cross combinations show almost the same LD attenuation pattern. LD declined to half of its peak value around 10 kb and approached the baseline at approximately 100 kb. Based on the LD decay map, we determined the upper/downstream annotation range of the significance SNPs. Candidate genes were labeled within half the physical distance (i.e., 10 kb) of the upstream and downstream LD decays at significant SNP sites. Given the relative lack of annotation information for QTLs, candidate QTLs were labeled within the physical distance (i.e., 100 kb) of upstream and downstream LD decay to a baseline at significant SNP sites.

### 3.3. Estimation of Genetic Parameters

The Supplementary Tables S1–S6 describe the significance of the fixed-effect factors listed in Table 1 with respect to the traits. Table 3 displays the calculated genetic parameters genetic parameters for six body weight traits in crossbred sheep, derived from a multi-trait model by Hiblup. For comparison, the heritability calculated using the single-trait model in HIBLUP is presented in Supplementary Table S7.

### 3.4. Genome-Wide Association Studies

**Figure 3:**
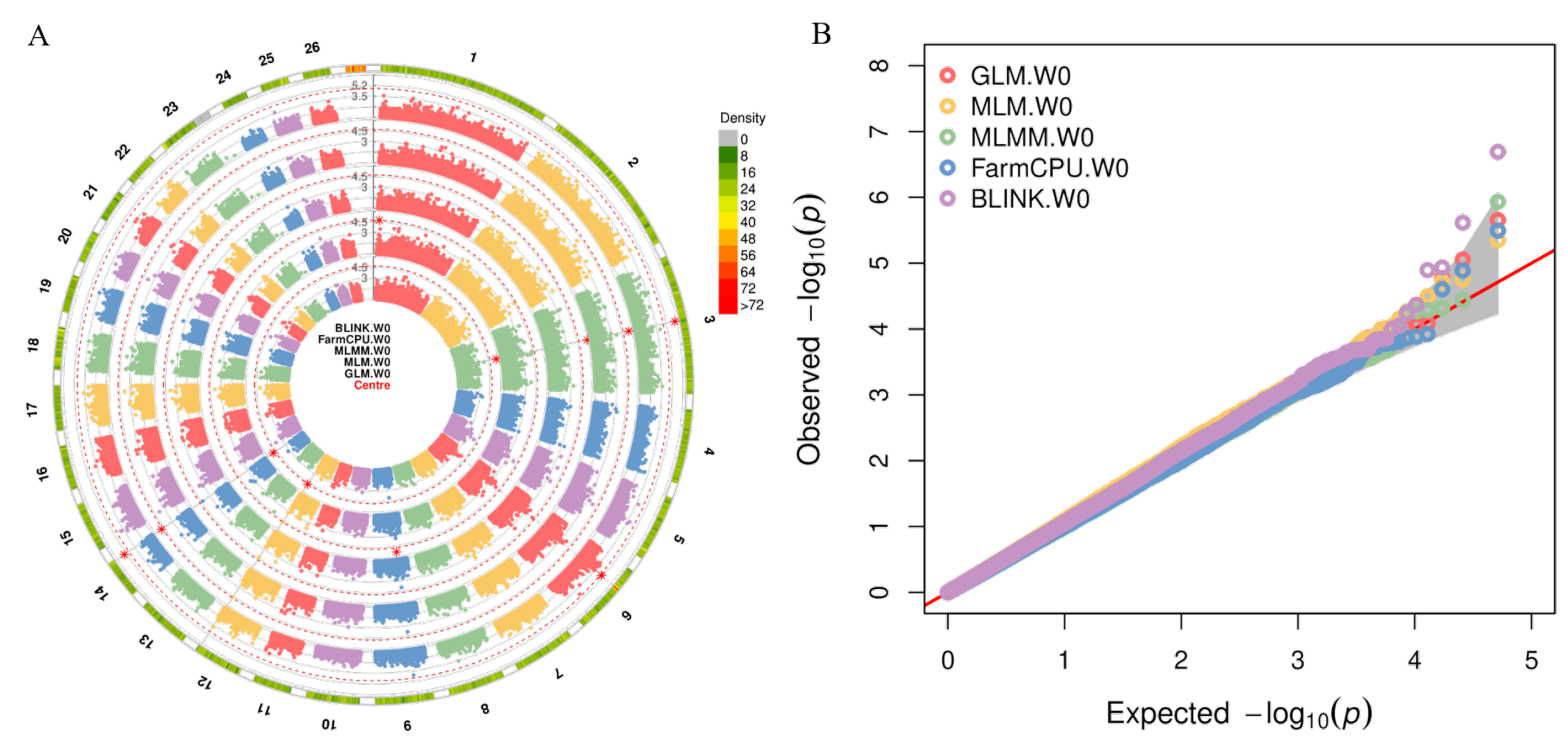
Some GWAS results for W0 traits are visually presented here. (**A**) represents a circular Manhattan plot from GWAS results, where the red asterisk marks all statistically significant SNPs; (**B**) shows a Q-Q plot from GWAS results, where the gray shaded area indicates the confidence interval.

Using birth weight (W0) as an example, Figure 3A shows the circular Manhattan plot of the W0 trait, where the outermost ring is the SNP density map and the inner layers are composed of various rings that display the outcomes of multiple models analyzing the identical trait. The red dotted line delineates the threshold for genome-wide significance, while the red asterisk indicates all significant SNPs. Circular Manhattan plots reveal strong associations between certain SNPs, such as SNPs on chromosomes 3 and 14, and W0, identifying these SNPs as potential key loci. The consistent detection of these SNPs across various models bolsters the validity of their association with the traits. Additionally, multiple Q-Q plots (Figure 3B) for trait W0 demonstrate that Q-Q scatter plots for all models align closely with the 45-degree diagonal line up to a *p*-value threshold of $10^{-3}$, confirming the expected uniform distribution of data without notable deviations. Beyond this threshold, however, all models show upward trends in the scatter plots, signifying significant associations between detected SNPs and the traits, thus identifying these SNPs as likely genuine key genetic markers. The circular Manhattan plot and Q-Q plot for the other traits are provided in Supplementary Figures S1–S12. A total of 48 significant SNPs were identified by these models, of which 7, 9, 14, 24, 2 and 1 SNP(s) were associated with W0, W30, W60, W90, W180 and W360, respectively. Significant SNPs were located on chromosomes 1, 2, 3, 6, 7, 8, 10, 13, 14, 18, 19, 22, and 23. Among them, the largest number of significant SNP sites related to the target traits were detected on chromosomes 6, 1, and 10. The GIF calculated by all models are presented in Supplementary Table S14. The results show that the GIF values for the GLM and MLM models significantly deviate from 1, whereas the values for the MLMM, FarmCPU, and BLINK models remain close to 1.

### 3.5. E-GWAS Strategy Integrated Multiple GWAS Models

**Figure 4:**
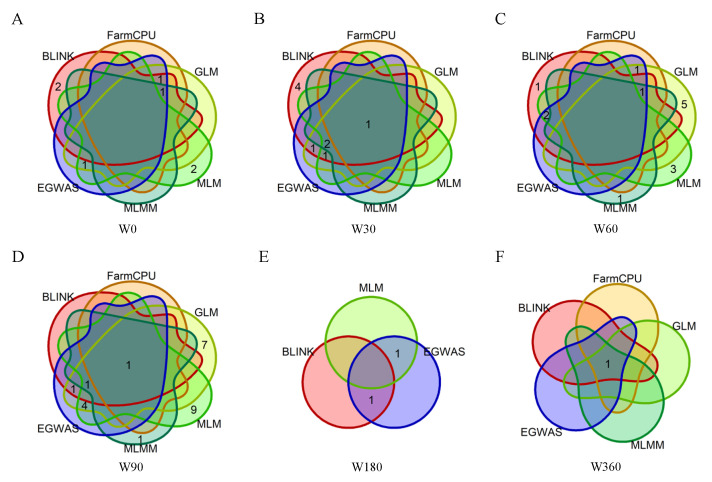
(**A**–**F**) Venn diagrams display SNPs identified by multiple GWAS models and the E-GWAS model for W0-W360.

Figure 4 displays the intersection of significant SNPs identified by five GWAS models and the E-GWAS strategy. In traits W60 and W90, E-GWAS omitted 50–70% of the SNPs identified by GLM and MLM. This high exclusion rate implies that GLM and MLM may exhibit elevated false-positive rates, corroborating findings reported by GIF. More specific E-GWAS results are presented in Supplementary Table S15.

### 3.6. Post-GWAS Analysis Using Various Databases

This post-GWAS analysis based on databases identified 24 genes, 4 of which were previously uncharacterized and considered potential determinants of body weight traits in the study. Moreover, the 100kb upstream and downstream regions of these significant SNPs overlap with nine QTLs previously linked to traits including body weight, as well as milk fat yield and milk production.

**Figure 5:**
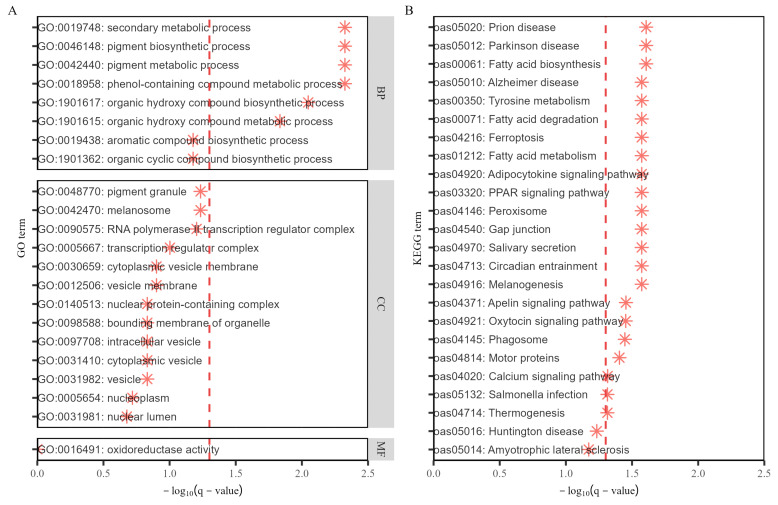
(**A**,**B**) displays the outcomes of GO and KEGG analyses, respectively. The vertical axis is labeled with GO and KEGG identifiers alongside specific terms, while the horizontal axis represents the negative base-10 logarithm of the q-values. A red dotted line denotes the 0.05 threshold of *q*-value. Red asterisks highlight the negative logarithms of *q*-value corresponding to the GO or KEGG terms.

Both GO and KEGG enrichment analyses were performed for genes with official names. The GO analysis identified 22 annotations related to gene-reactive graphene oxide, whereas the KEGG analysis identified 24 biological pathways associated with five genes. Employing a significance threshold for q-values set at 0.05, 6 GO annotations and 22 biological pathways achieved significance. Figure 5A,B display the results of the GO and KEGG analyses, including annotation numbers and descriptions. The analysis demonstrates an over-representation of the target gene set in specific GO or KEGG terms relative to the background set. Greater abundance is generally associated with elevated significance levels, where statistical correlations for each abundance are established by q-values below 0.05, signifying both statistical significance and biological relevance. GO annotations were broadly classified into four categories: metabolic processes and biosynthesis, cellular structures and tissues, enzyme activity, and transcriptional regulatory complexes. Additionally, in KEGG enrichment analysis, annotations were categorized into five main areas: metabolic processes, signal transduction pathways, immune response, neurodegenerative diseases, and physiological functions. All results derived from database annotations are presented in Supplementary Tables S16–S19.

## 4. Discussion

### 4.1. Influencing Factors

This investigation examined the impact of various non-genetic influences on weight at different developmental stages of lambs. The significance of fixed effects on specific traits, as shown in Supplementary Tables S1–S6, reveals a significant influence of all fixed effects, with the exception of the hybrid group for the W0 trait, which demonstrates no significant effect.

Our results indicate that gender significantly affects weight at all stages, with male lambs generally weighing more than females. This difference may be attributed to physiological and genetic variations that promote greater weight accumulation during growth. These findings align with the conclusions drawn in studies of Kermani sheep by Bahreini Behzadi et al. [29] and Makuie sheep by Rahimi et al. [30]. Benyi et al. suggest that faster intrauterine growth rates in male lambs might explain their higher birth weights [31]. Hormonal differences, particularly the elevated testosterone production in males, which acts as a growth promoter, could explain the diverging weight trajectories between genders as lambs age [32].

Birth type also plays a crucial role in weight traits, with singletons typically weighing more than twins. Singletons likely receive more nutrients and space for growth in the uterus. Sharif explains that throughout the lactation phase, the restricted capacity for milk production in ewes could result in lower weaning weights for twins due to competitive behaviors [33]. In contrast, singletons receive full maternal care and optimal nutrition [33].

The age of the ewe significantly influences lamb weight throughout gestation and lactation phases. Supporting evidence from other studies confirms the substantial impact of ewe age on pre-weaning growth traits, aligning with these results [30,34]. Older ewes typically provide a superior uterine environment and exhibit stable reproductive capabilities. This factor continues to influence lamb weight traits at stages W30, W60, and W90, primarily reflecting colostrum quality and effective care management, which are critical for early growth. Consequently, lambs from older ewes exhibit faster and more robust growth, with a pronounced upward trend in weight gain [33].

From a husbandry perspective, different rearing groups and management practices significantly influence weight traits post-birth. Feed quality, frequency, and methods are vital for optimal growth rates in lambs. Shi et al. [35] states that lambs raised in different conditions under various managers with unique rearing programs and techniques exhibit diverse growth outcomes due to these varied practices.

Hybrid group also influences weight traits, where crossbred sheep tend to weigh more than non-crossbreds post-weaning, a likely result of the genetic potential of different crossbreeds [36,37]. In a prior comparative study on the early growth performance of hybrid breeds between Texel and Kazakh sheep, researchers observed significant variations in growth trends across different hybrid combinations [38]. While crossbreeding has a minimal impact at birth in this study, its significance becomes apparent in later weight traits.

The research indicates that, in addition to genetic factors, non-genetic factors significantly influence lamb weight variation. Although the impact of these factors varies across different growth stages, they collectively exert a substantial effect on lamb weight. This finding suggests that optimizing the use of these non-genetic factors could effectively enhance the weight traits of sheep.

### 4.2. Genetic Parameters

This study evaluated the genetic parameters of hybrid breeds between Texel and Kazakh sheep, encompassing heritability, genetic correlation, and phenotypic correlation. Both the single-trait and multi-trait models from Hiblup were used to calculate heritability and genetic correlation coefficients. These models facilitated the estimation of genetic contributions for each trait. Heritability estimates within the single-trait model varied from 0.07 (W180) to 0.40 (W90), whereas in the multiple-trait model, these estimates ranged from 0.11 (W360) to 0.22 (W90). The multi-trait model results, deemed more reliable, integrate data from multiple traits and consider their inter-relationships. Additionally, these results align with findings from previous studies [39,40,41], reinforcing their validity.

Multi-trait model surpass single-trait model in estimating heritability and also enable the assessment of genetic correlations, which are unattainable with single-trait models. The genetic correlation coefficients derived from multi-trait model range from 0.26 to 0.98. These positive values indicate that genetic linkages between traits vary from moderate to high. Typically, the genetic gain in one trait corresponds to the genetic gain in another, reflecting a positive genetic correlation.

The phenotypic correlation coefficients between weight traits, specifically the Pearson correlation coefficients, ranged from 0.36 to 0.91, indicating moderate to high positive correlations. These correlations may reflect a shared genetic background among the traits and suggest the influence of similar environmental or physiological conditions. Consistently observed positive correlations indicate that individuals with higher weights tend to maintain this weight in subsequent measurements.

Accurate assessment of heritability and genetic correlations is crucial for the effective design of sheep breeding programs. Although direct selection for traits with medium to high heritability can lead to rapid genetic gains, optimizing environmental and management practices is crucial for sustaining long-term genetic improvement in traits with low heritability. Additionally, utilizing genetic correlations through indirect selection could be a viable approach to enhance multiple weight traits simultaneously, thereby accelerating the overall productivity of sheep.

### 4.3. Model Comparison

To assess the performance of various GWAS models, this study analyzed five models: GLM, MLM, MLMM, FarmCPU, and BLINK. The evaluation involved Q-Q plots and GIF for six traits (W0, W30, W60, W90, W180, and W360). Q-Q plots facilitated the visual assessment of *p*-value distribution. Except for the GLM and MLM models, which deviated significantly from the 45-degree reference line for certain traits (W30, W60, and W90), other models closely followed this reference line from the origin to $10^{-3}$ across all traits.

For a more objective comparison, the genome inflation factor for each model on the target traits was calculated. The results indicated that both the GLM model, with a GIF ranging from 1.074 (W180) to 1.4087 (W90), and the MLM model, with a GIF ranging from 1.0116 (W180) to 1.3461 (W90), consistently showed higher GIF values, suggesting significant effects from genome inflation and population stratification. In contrast, the other three models performed well across different traits. Notably, the mean GIF values for MLMM was closest to 1, ranging from 0.9934 to 1.0187, effectively controlling for population stratification and genome inflation. FarmCPU and BLINK exhibited optimal performance on specific traits. The GIF values of FarmCPU were slightly below 1, from 0.9305 (W90) to 0.9966 (W360), indicating potential slight overcorrection and power loss. Although the GIF values of BLINK were close to 1, they were slightly higher than those of MLMM in some traits (e.g., GIF of 1.0887 for W0 and 1.0408 for W180), suggesting reasonable but sometimes suboptimal control of genome inflation. Regarding computational speed, GLM was the fastest, followed by BLINK and FarmCPU, while MLM and MLMM were slower.

Overall, GLM exhibited the poorest performance across all models, potentially leading to an excess of false positives. In contrast, the MLM outperformed the GLM. By comparison, the other three models demonstrated balanced performance on this dataset, with each model excelling in at least one trait.

### 4.4. Post-GWAS and Gene Annotation

This study utilized E-GWAS to integrate outcomes from five distinct GWAS models. Unlike simple aggregations of results, E-GWAS applies stacking ensemble techniques to refine SNP selection, enhancing the robustness of the resultant SNP list. The selection of the E-GWAS approach was prompted by the analysis of a medium-sized dataset, which is susceptible to significant outcome variability across different GWAS methodologies. Thus, the integration of multiple GWAS methods via E-GWAS is essential to comprehensively address target traits with varying genetic characteristics.

This study identified multiple candidate genes linked to target traits. Notably, certain genes are associated with several traits. Specifically, the genes *GLIS1* and *LOC105603112* are associated with W30, W60, and W90; *LRP1B* and *LOC101111090* correlate with W30 and W90; *GPC5* and *RYR3* are linked to W60 and W90. These findings support the hypothesis of pleiotropy in these regions. The traits W30, W60, and W90 represent lamb weights from birth to weaning, suggesting that these gene locations may form pleiotropic zones critical for lamb growth traits. Apart from the significant SNP identified at −142,634 bp on chromosome 22 for W360, the mapped gene is not a candidate gene. In contrast, other traits each possesses at least one significant SNP associated with a recognized gene, serving as a candidate gene.

In the present study, a notable overlap was observed between the 100-kilobase intervals upstream and downstream of the significant SNPs identified and certain previously documented QTL intervals. These QTLs were associated with nine traits such as Body weight, Total lambs born, Milk yield. Intuitively, some of these traits are directly or indirectly related to the target traits studied in this paper.

A GWAS research of the Jalgin Merino sheep breed identified the *CNTN3* gene as a potential gene linked to productivity [42]. In another GWAS research, Chen et al. explored the genetic foundations of neurotransmitter concentrations in cattle and identified the *CNTN3* gene as potentially related to neurotransmitter levels [43]. Yao et al. investigated the molecular mechanisms behind wool color in native Xinjiang sheep breeds in China, identifying the *DCT* gene as a potential regulator of color development [44]. Another study indicates that the expression of the *DCT* gene in sheep tanycytes varies seasonally. Influenced by photoperiods and thyroid hormones, these variations suggest the role of *DCT* in protecting against oxidative stress or controlling the proliferation of neural progenitor cells, deviating from conventional melanin synthesis pathways [45]. Located on chromosome 6 in sheep, the *FAM184B* gene is linked to meat quality, body weight, and conformation traits [46,47], as well as fat metabolism and skeletal development [1]. In Chinese fat-tailed sheep, the *GLIS1* gene correlates with fat accumulation in the tails, as demonstrated by whole-genome sequencing analyses of Mongolian, Small-tailed Han, and Thin-tailed Milk sheep breeds [48]. Additionally, resequencing data from Russian Tuva sheep supports this association [49]. In a variant calling analysis of 5061 sheep, the study used the GATK and Freebayes algorithms along with a Poisson model to address sequencing errors. This method revealed low-frequency variants in the *GPC5* gene associated with teat number in sheep [50]. In genetic comparisons of Afec-Assaf and Awassi sheep, the *GRB14* gene was located close to a variant-rich area on sheep chromosome 2. This region contains a few synonymous mutations, which are associated with fat distribution [51]. A GWAS study on the weight traits of sheep in Inner Mongolia, China, identifies *LRP1B* as a potential gene associated with birth weight [52]. Concurrent research by Martins et al. on Nelore cattle associated the *LRP1B* gene with rump fat thickness and proposed its function as a carrier for apolipoprotein E, a protein involved in fat accumulation in multiple species [53]. Ramos et al. executed a GWAS in Uruguayan Merino sheep utilizing a single-step Bayesian methodology, identifying *MED28* as a candidate gene for live weight traits [54]. In an independent analysis, a weighted single-step GWAS of Valle del Belice sheep identified *MED28* as one of the candidate genes for somatic cell score [55]. Researchers conducted a GWAS on three Indian sheep breeds using data from a 50K SNP chip [56]. The analysis of Runs of Homozygosity revealed significant homozygosity in the *NPHP1* gene region of the Deccani breed, suggesting a potential association with the selection of specific genetic traits or breeding history [56]. Studies investigating resistance in local Florida sheep to Haemonchus contortus identified an association between the *PCDH7* gene and the resistance phenotype in lambs against this parasite [57]. In a study using recombinant inbred mouse strains, the *PTPRK* gene is implicated in modulating the relative abundance of T cells and B cells, suggesting an association with genetic variability in immune function [58]. In Karachai goats, this gene correlates with chest girth and co-regulates intracellular fatty acid metabolism alongside other genes involved in diverse metabolic pathways [59]. In a GWAS of Esme lambs, the gene *PTPRT* emerged as a potential marker linked to pre-weaning growth traits [60]. Analysis of 600K high-density SNP data from sheep samples across 13 populations revealed associations of the *RYR3* gene with fat deposition in sheep tails [61]. Moreover, Antkowiak et al. identified frequency changes in structural variations near the *RYR3* gene, distinguishing obese from healthy Labrador Retrievers through structural variation analysis [62].

Research by Xiao et al. indicates that polymorphisms in the *ACSL5* gene serve as pivotal genetic determinants of meat quality and carcass yield in Chinese Simmental crossbred beef cattle [63]. Pang et al. reported that the *DCLK1* gene is upregulated during overexpression of the bovine papillomavirus E6 gene, indicating a potential role in immune-related processes [64]. Li et al. performed Mendelian randomization analyses to investigate the potential causative link between *EFCAB14* and human osteoarthritis, finding a significant negative correlation that suggests *EFCAB14* may inhibit disease development [65]. A study employed the ComBat method to integrate gene expression data associated with presenile dementia and identified five genes, including *KIAA0513*, related to the disease through weighted gene co-expression network analysis in a mouse model [66]. The human protein complex map, developed by Drew et al. using data from multiple mass spectrometry experiments, predicted and experimentally confirmed the role of *KIAA1328* as a novel centriolar/ciliary satellite protein in vertebrate models [67]. Overexpression of the *TUBB6* gene disrupts microtubule networks in muscle fibers of Duchenne Muscular Dystrophy mouse models, analogous to pathologies observed in mdx mice, indicating its pivotal role in microtubule organization [68]. Moreover, knockout studies by Maurin et al. employing CRISPR/Cas9 in osteoclasts reveal that *TUBB6* likely regulates *CDC42* activity through modulation of ARHGAP10-microtubule interactions, thereby facilitating bone resorption [69]. In obese mice, the downregulation of LEPR and IGFBP1 mediated by *ZBTB20* contributes to lipid accumulation and inflammation, respectively [70]. Research employing overexpression and knockdown strategies has demonstrated that *ZBTB20* regulates astrocyte differentiation in the neocortex. It promotes astrocytogenesis during neocortical development through synergistic interactions with Sox9 and NFIA and by repressing *BRN2* expression [71]. Rajawat et al. utilized statistical selection signatures from eight distinct features to identify key genomic footprints in cattle breeds, establishing the *ZBTB20* gene as a candidate influencing factor for reproductive traits [72].

## 5. Conclusions

This study employed a variety of GWAS models and the E-GWAS strategy, leveraging various databases to elucidate the genetic characteristics of body weight in hybrid offspring of the globally dominant Texel breed and Chinese Kazakh sheep. GWAS identified 48 significant SNPs annotated to 20 genes, including *ACSL5*, *CNTN3*, *DCLK1*, *DCT*, *EFCAB14*, *FAM184B*, *GLIS1*, *GPC5*, *GRB14*, *KIAA0513*, *KIAA1328*, *LRP1B*, *MED28*, *NPHP1*, *PCDH7*, *PTPRK*, *PTPRT*, *RYR3*, *TUBB6*, *ZBTB20*, and 4 uncharacterized genes. Chromosome 6 harbored the highest number of related genes, significantly influencing the body weight of sheep. The post-GWAS analysis implemented the E-GWAS strategy, which integrated findings from five distinct GWAS models to generate a stable list of SNPs, rather than merely compiling a simple aggregate of SNP lists. To further elucidate the relationships between genetic variants and phenotypic traits, the study incorporated data from multiple databases. Annotation analysis incorporated data from various databases to enhance the comprehension of the links between genetic variations and phenotypic traits. The analysis identified 9 QTLs, 6 GO annotations, and 22 KEGG annotations. Future research should integrate experimental methods to explore body weight or other traits, such as parasite resistance, disease susceptibility, and wool characteristics, uncovering the complex molecular mechanisms behind these traits to design more comprehensive and effective sheep breeding programs, enhancing productivity, profitability, and sustainability.

## Figures and Tables

**Table 1:** Inclusion of factors as fixed effects at different traits.

Factor	W0	W30	W60	W90	W180	W360
Hybrid Group	Y	Y	Y	Y	Y	Y
Rearing Group	N	Y	Y	Y	Y	Y
Birth Type	Y	Y	Y	Y	Y	Y
Gender	Y	Y	Y	Y	Y	Y
Age of Ewe	Y	Y	Y	Y	N	N

Y: Add this effect; N: do not add this effect.

**Table 2:** Descriptive statistics of phenotypes.

Trait	Number	Mean	SD	Median	Min	Max	Skew	Kurtosis	IQR
W0	573	5.21	0.86	5.20	2.90	7.30	−0.18	−0.34	1.20
W30	569	12.37	2.19	12.47	6.52	18.29	−0.18	−0.15	2.96
W60	561	19.76	3.47	19.97	10.76	28.70	−0.22	−0.28	4.41
W90	568	28.25	4.71	28.44	15.21	41.12	−0.11	−0.07	6.45
W180	493	39.08	5.15	39.19	25.24	52.15	−0.08	−0.24	6.83
W360	488	45.73	7.24	45.49	25.99	65.67	−0.01	−0.20	9.77

**Table 3:** Heritability (bold on diagonal), genetic correlation (below diagonal), phenotypic correlation (above diagonal), and standard error (in parentheses).

	W0	W30	W60	W90	W180	W360
**W0**	0.13(0.17)	0.73(0.03)	0.65(0.03)	0.67(0.03)	0.49(0.04)	0.39(0.04)
**W30**	0.26(0.71)	0.17(0.15)	0.82(0.02)	0.82(0.02)	0.60(0.04)	0.45(0.04)
**W60**	0.43(0.58)	0.52(0.40)	0.22(0.15)	0.91(0.02)	0.59(0.04)	0.36(0.04)
**W90**	0.66(0.54)	0.64(0.37)	0.85(0.16)	0.22(0.15)	0.64(0.03)	0.41(0.04)
**W180**	0.45(0.81)	0.84(0.39)	0.52(0.50)	0.51(0.49)	0.13(0.16)	0.67(0.03)
**W360**	0.37(0.83)	0.86(0.76)	0.50(0.62)	0.48(0.57)	0.98(0.57)	0.11(0.15)

## Data Availability

Data will be available upon request from the corresponding author.

## Supplementary Materials

The accompanying supplementary information can be accessed for download at: https://www.mdpi.com/article/10.3390/genes15121521/s1. Figures S1–S6: Circular Manhattan plot for body weight traits; Figures S7–S12: Q-Q plot for body weight traits; Tables S1–S6: ANOVA tables for covariates and fixed effects on various body weight traits. Table S7: The heritability of body weight traits calculated using a single-trait model; Tables S8–S13: The results of GWAS for body weight traits; Table S14: Calculated results of genome inflation factors (GIFs) for all models; Table S15: List of SNPs obtained by E-GWAS strategy; Tables S16–S19: The post-GWAS analysis results utilizing various databases.
