# Supplementary figures and images for: Genome-Wide Association Study of Body Weight Traits in Texel and Kazakh Crossbred Sheep
Source: Genes (Basel). 2024 Nov 27;15(12):1521. doi: 10.3390/genes15121521 (PMC11675303; doi:10.3390/genes15121521)

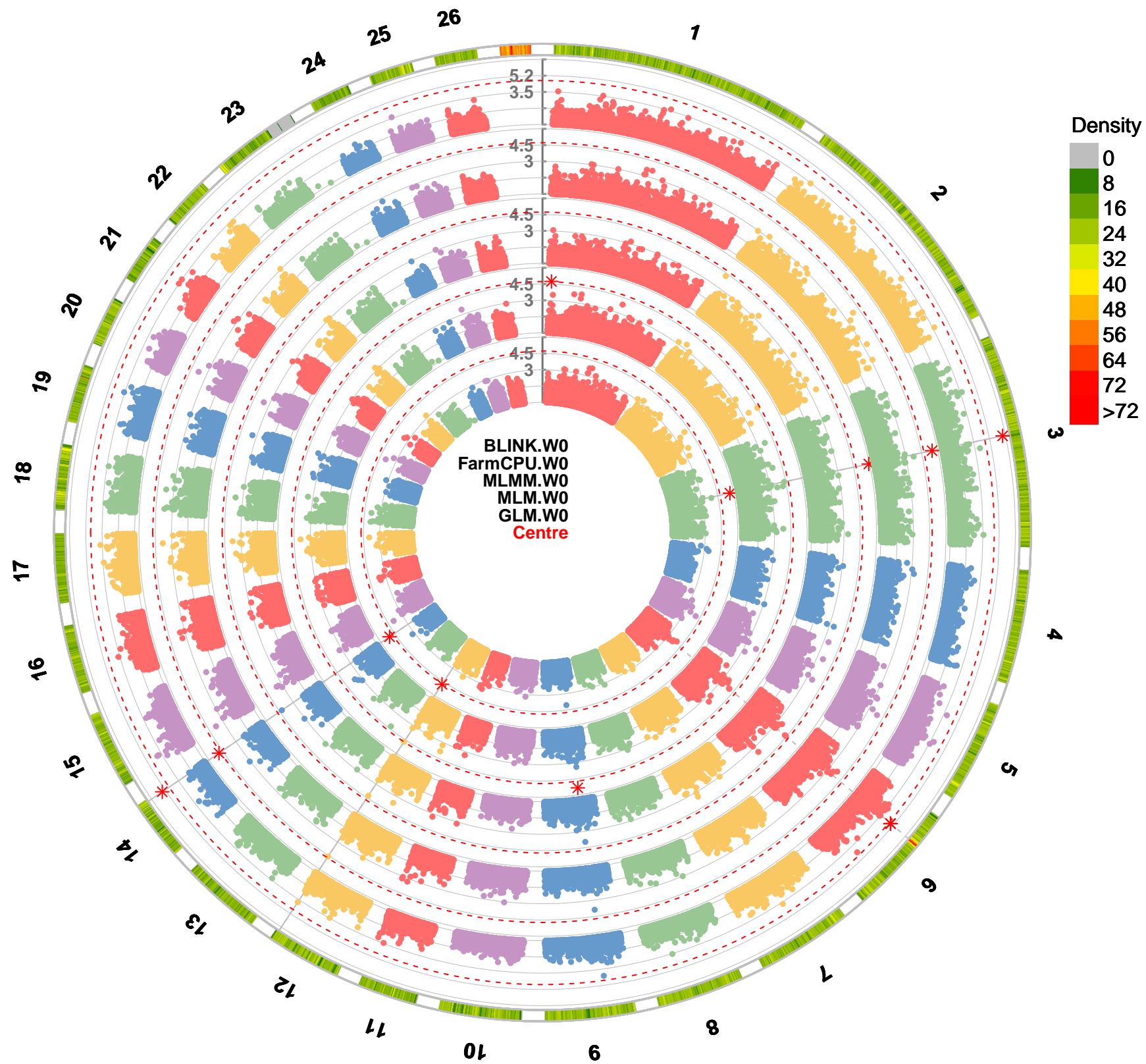

Supplement: Supplementary file 1 [file genes-15-01521-s001.zip › Figure S1.pdf]

QQ plot

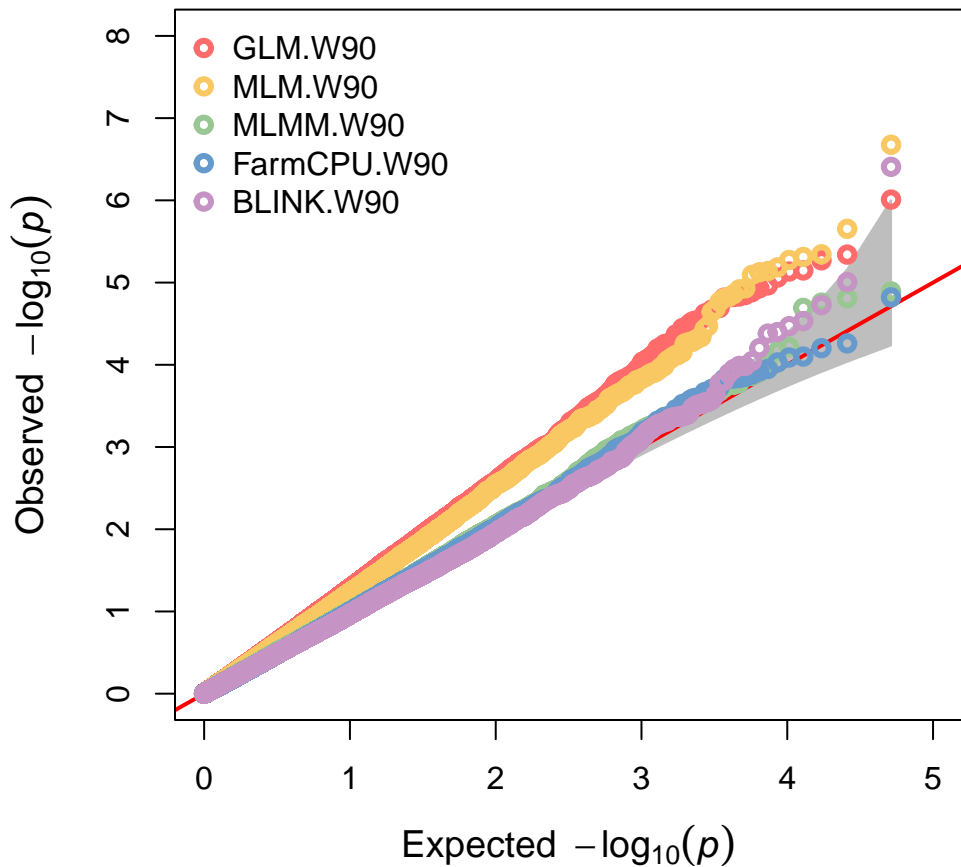

Supplement: Supplementary file 1 [file genes-15-01521-s001.zip › Figure S10.pdf]

# QQ plot

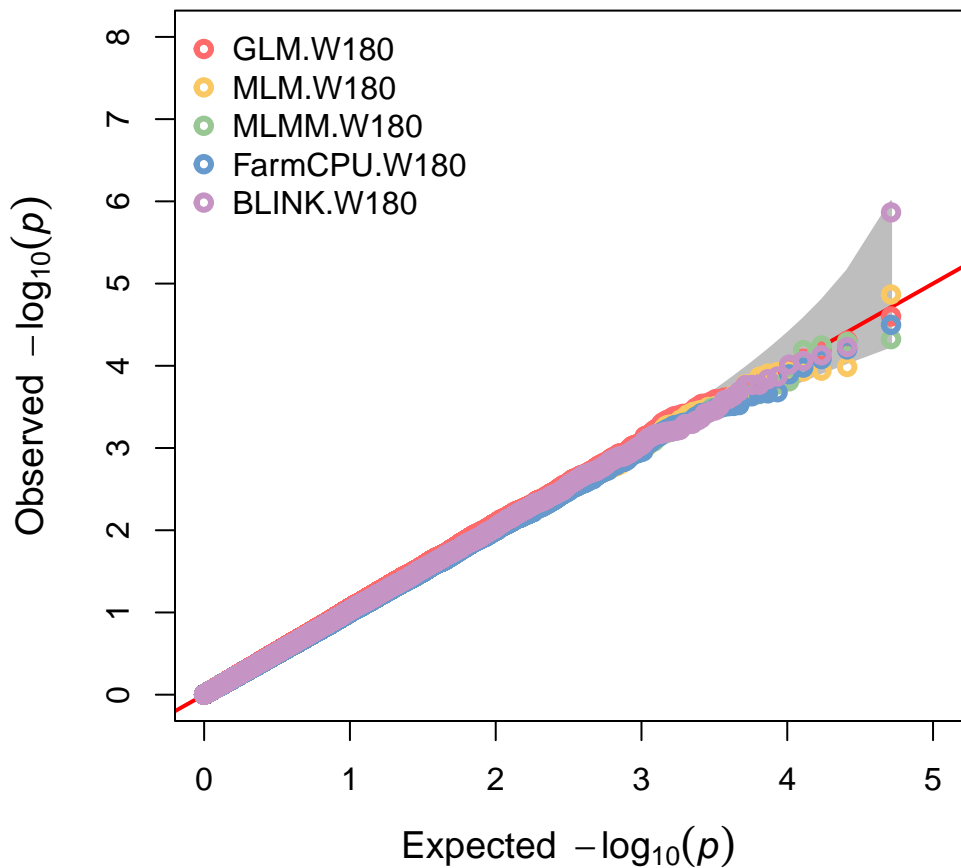

Supplement: Supplementary file 1 [file genes-15-01521-s001.zip › Figure S11.pdf]

# QQ plot

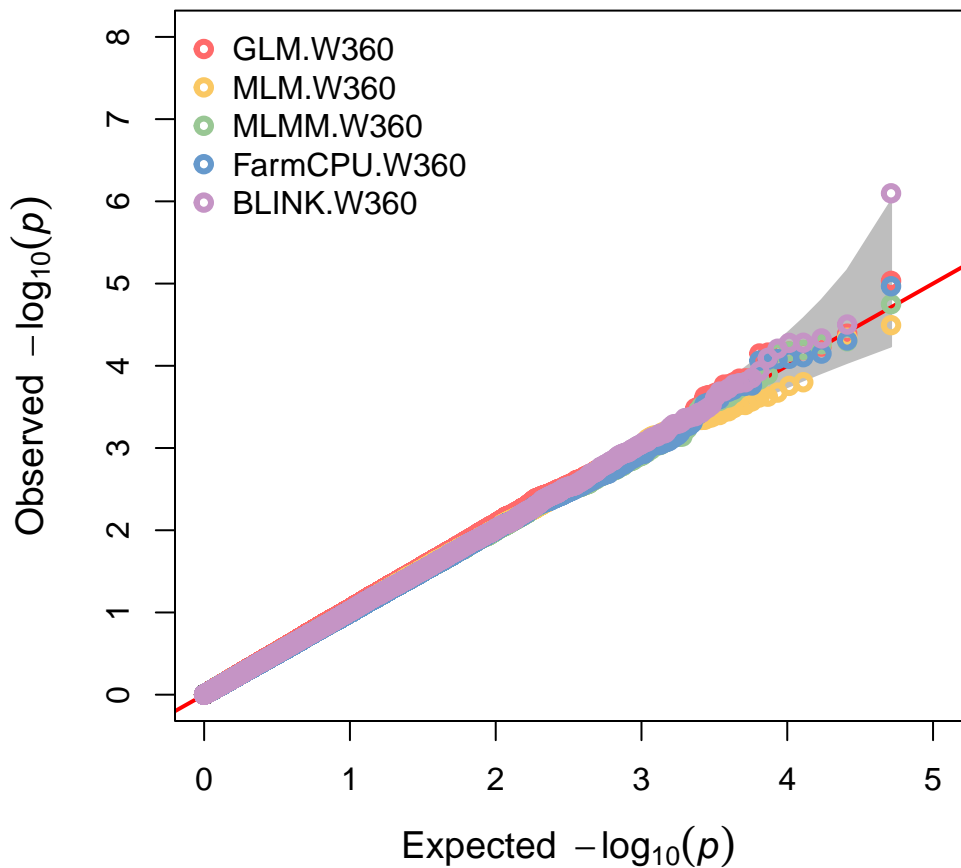

Supplement: Supplementary file 1 [file genes-15-01521-s001.zip › Figure S12.pdf]

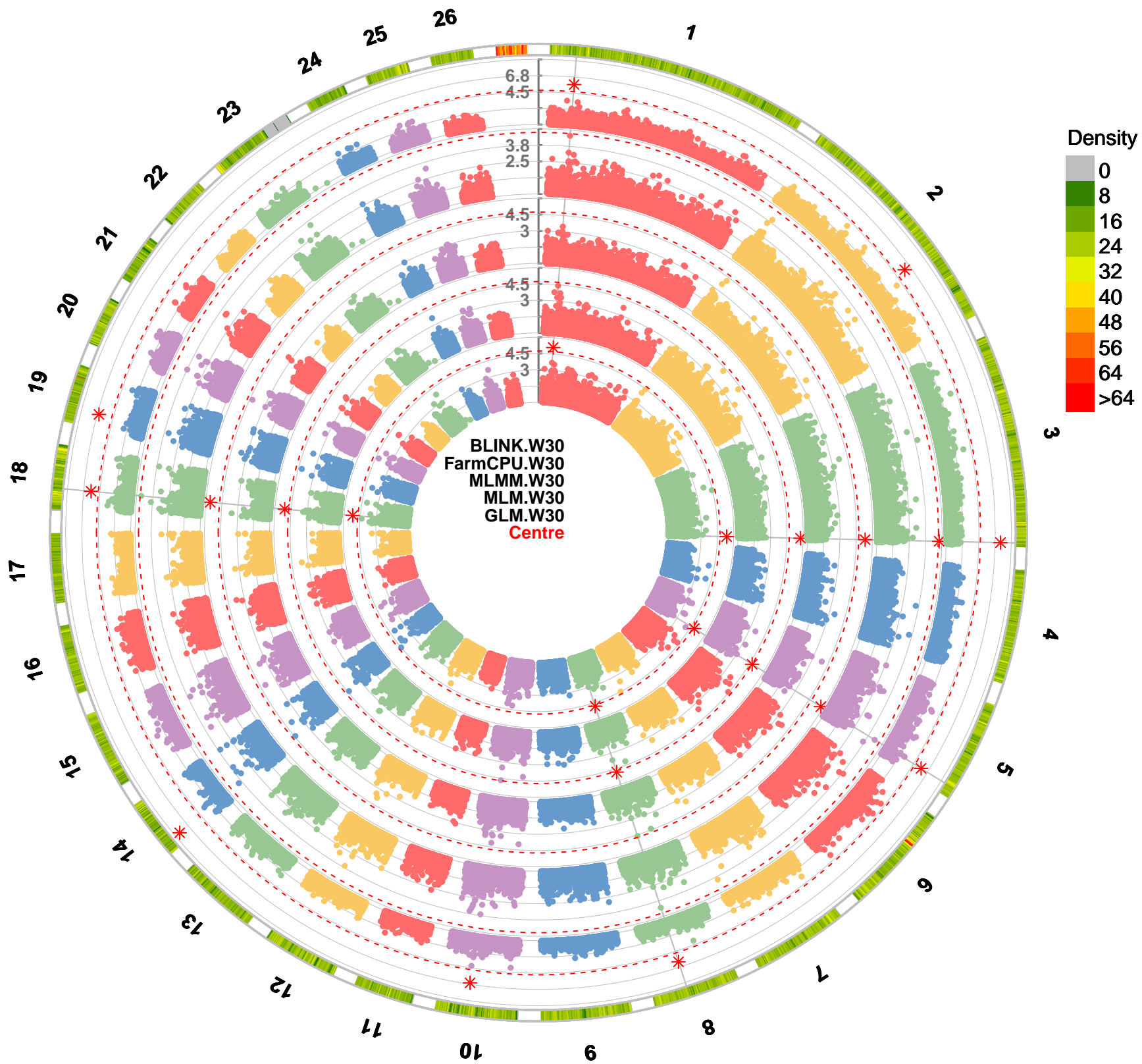

Supplement: Supplementary file 1 [file genes-15-01521-s001.zip › Figure S2.pdf]

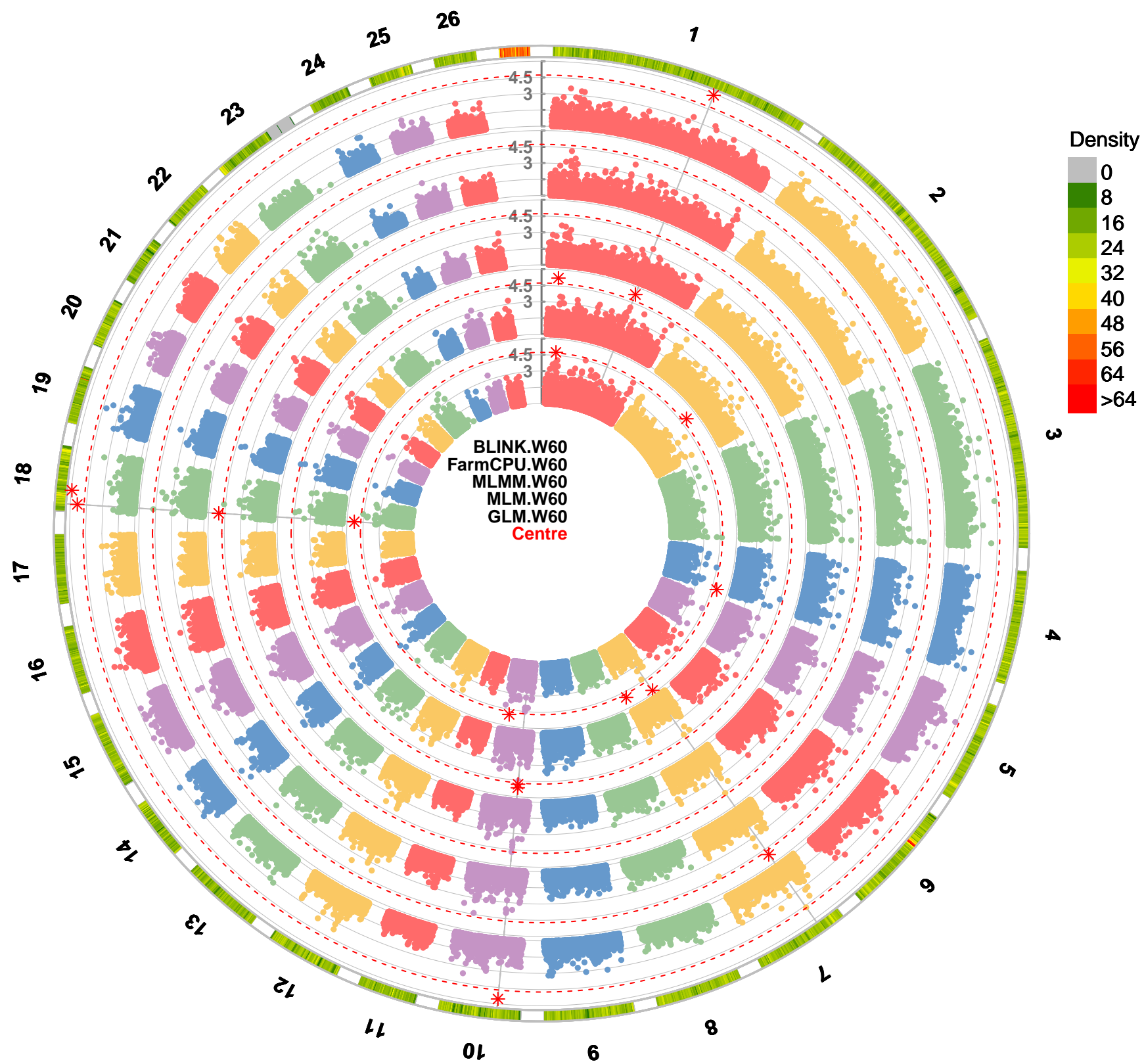

Supplement: Supplementary file 1 [file genes-15-01521-s001.zip › Figure S3.pdf]

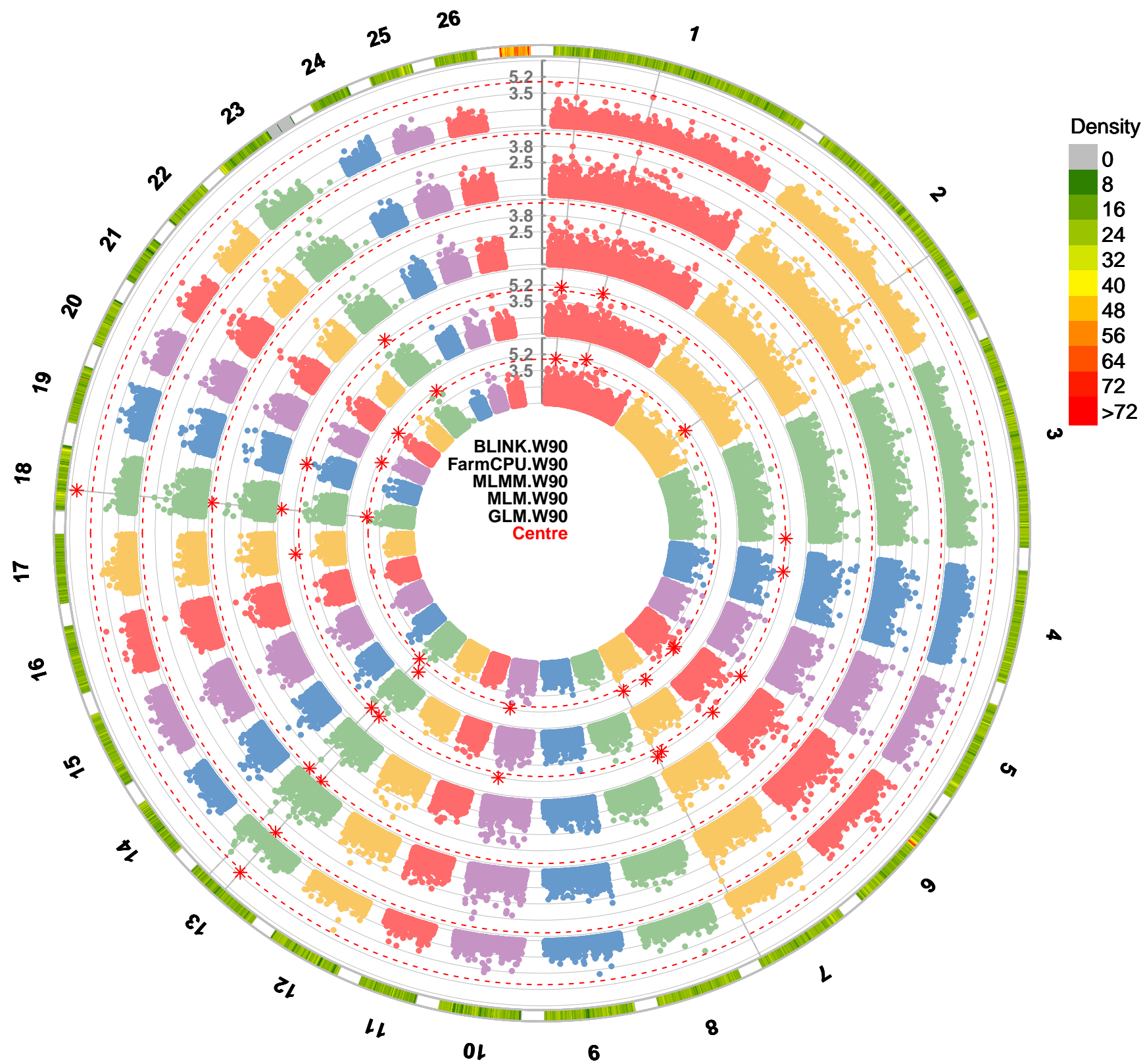

Supplement: Supplementary file 1 [file genes-15-01521-s001.zip › Figure S4.pdf]

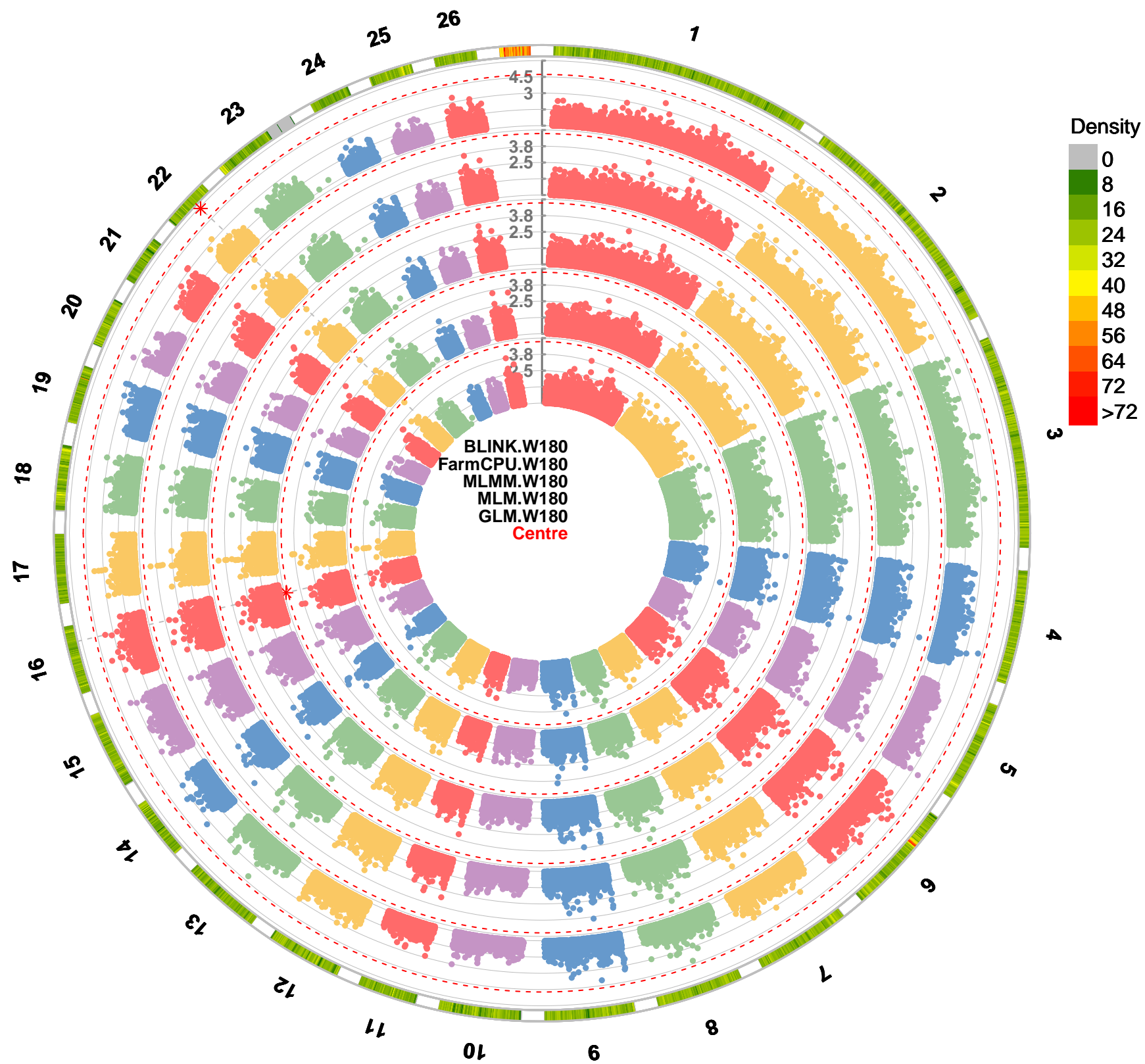

Supplement: Supplementary file 1 [file genes-15-01521-s001.zip › Figure S5.pdf]

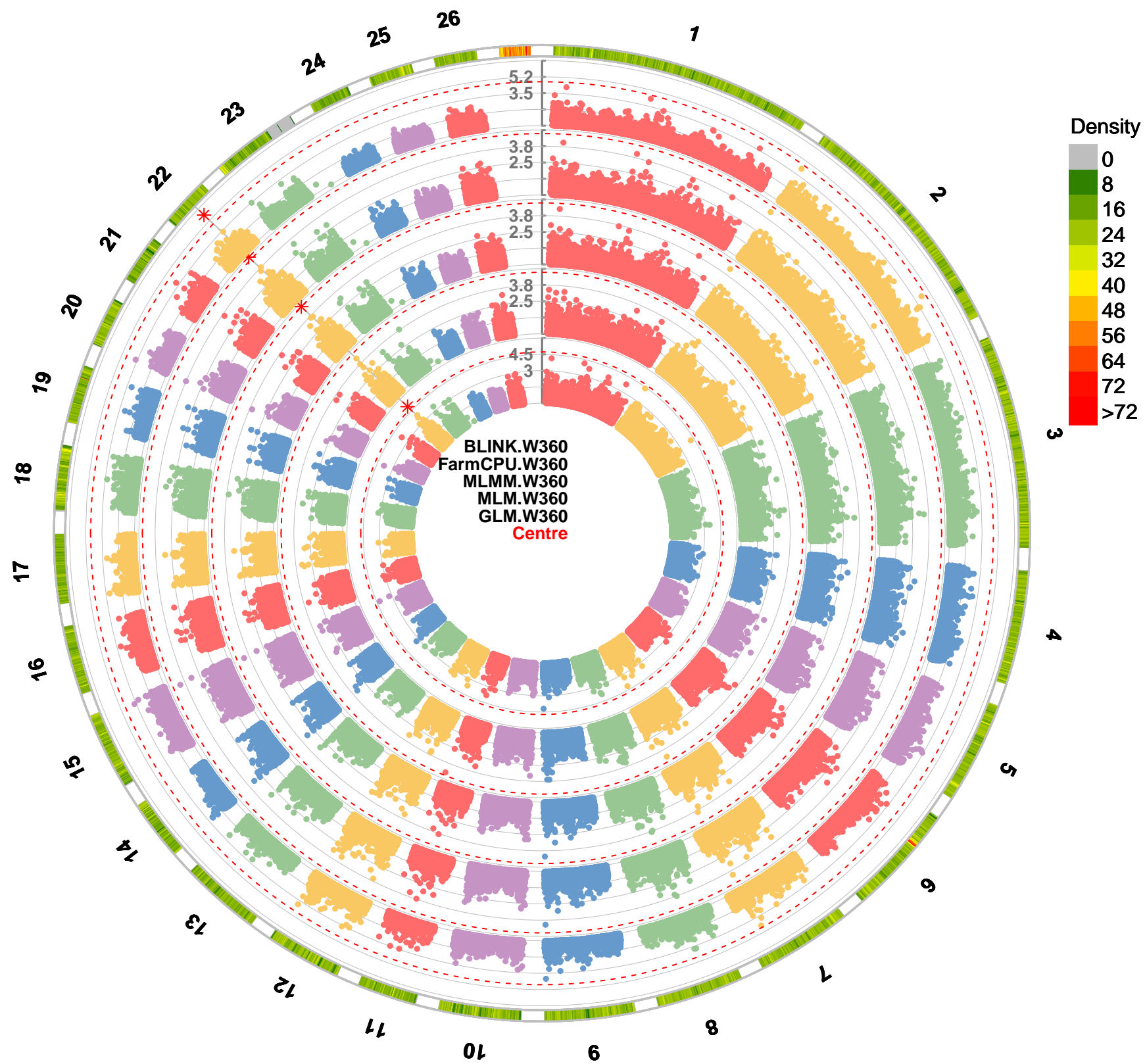

Supplement: Supplementary file 1 [file genes-15-01521-s001.zip › Figure S6.pdf]

QQ plot

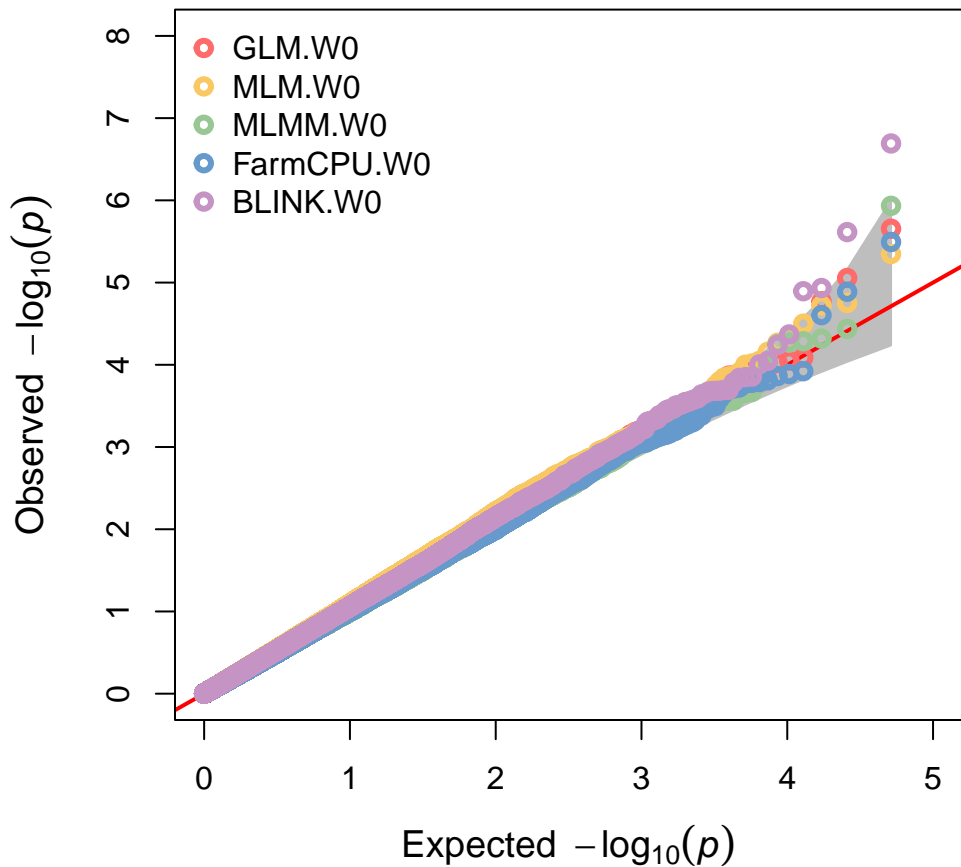

Supplement: Supplementary file 1 [file genes-15-01521-s001.zip › Figure S7.pdf]

QQ plot

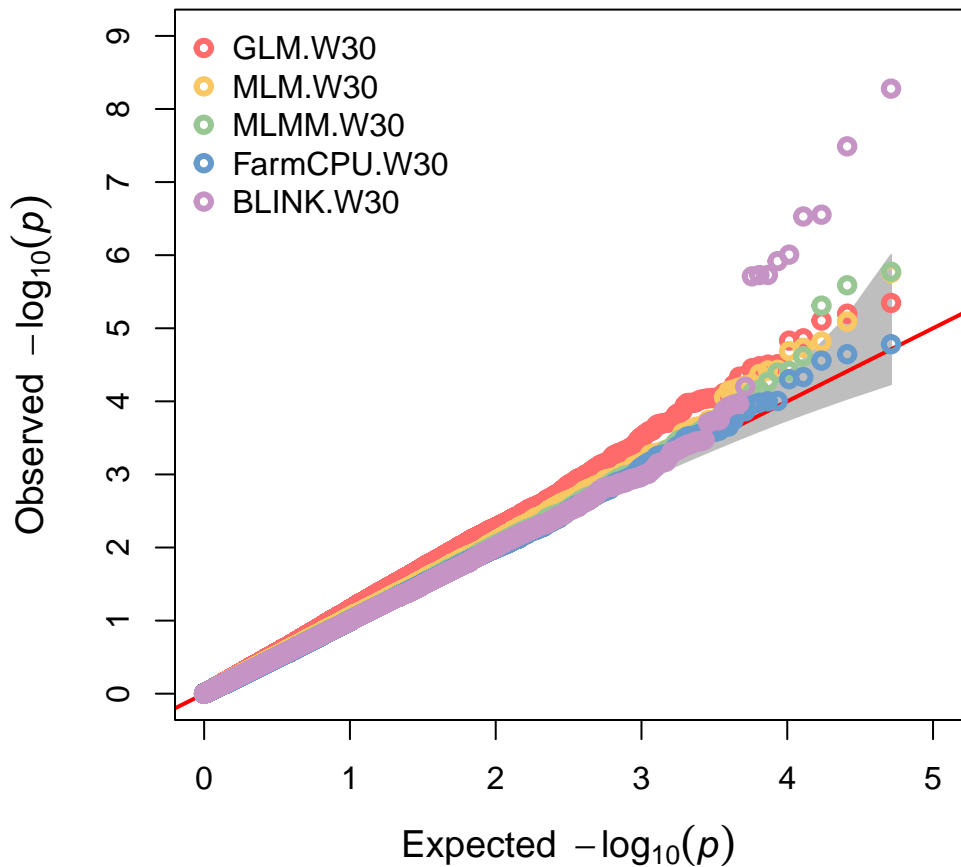

Supplement: Supplementary file 1 [file genes-15-01521-s001.zip › Figure S8.pdf]

QQ plot

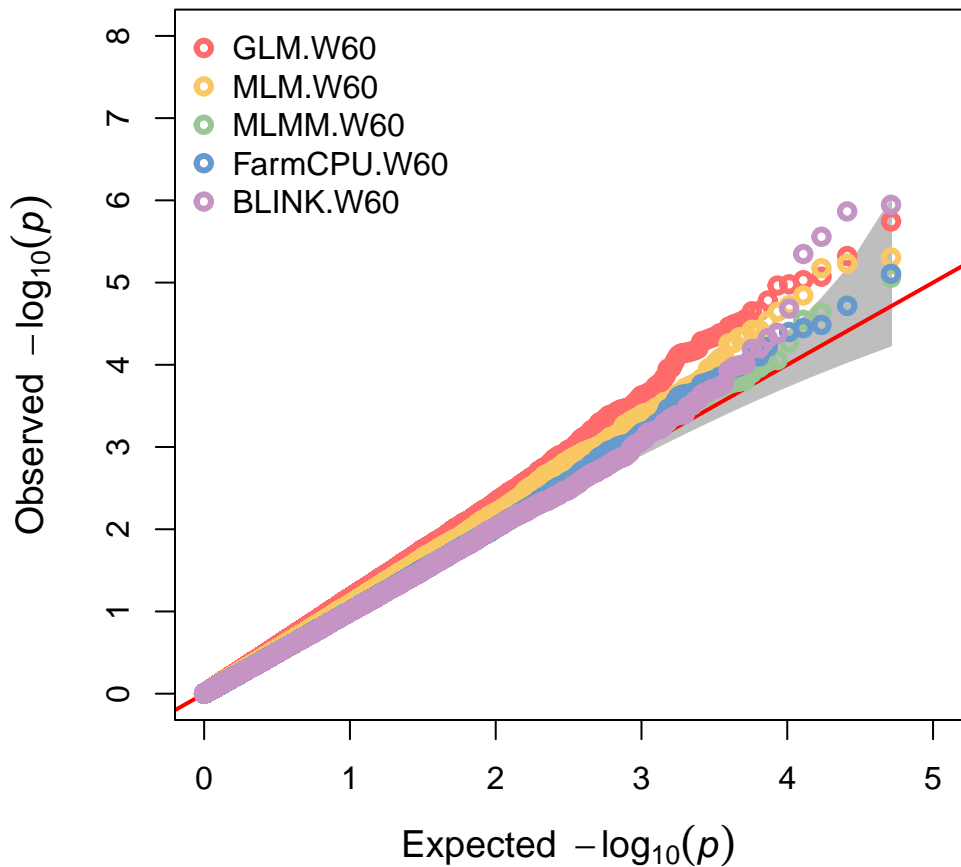

Supplement: Supplementary file 1 [file genes-15-01521-s001.zip › Figure S9.pdf]
